# Pulmonary pathology and COVID-19: lessons from autopsy. The experience of European Pulmonary Pathologists

**DOI:** 10.1007/s00428-020-02886-6

**Published:** 2020-07-09

**Authors:** Fiorella Calabrese, Federica Pezzuto, Francesco Fortarezza, Paul Hofman, Izidor Kern, Angel Panizo, Jan von der Thüsen, Sergei Timofeev, Gregor Gorkiewicz, Francesca Lunardi

**Affiliations:** 1 Department of Cardiac, Thoracic, Vascular Sciences and Public Health, University of Padova Medical School, Via A. Gabelli 61, 35121 Padova, Italy; 2 Laboratory of Clinical and Experimental Pathology, FHU OncoAge, Biobank BB-0033-00025, University Côte d’Azur, Nice, France; 3 University Clinic of Respiratory and Allergic Diseases, Golnik, Slovenia; 4 Complejo Hospitalario de Navarra, Pamplona, Navarra Spain; 5 Department of Pathology, Erasmus MC, Rotterdam, The Netherlands; 6 Moscow City Hospital #40, Moscow, Russia; 7 Institute of Pathology, Medical University of Graz, Graz, Austria

**Keywords:** COVID-19, SARS-CoV-2, Autopsy, Lung, Pandemic

## Abstract

Since its initial recognition in December 2019, Coronavirus disease 19 (COVID-19) has quickly spread to a pandemic infectious disease. The causative agent has been recognized as a novel coronavirus, severe acute respiratory syndrome coronavirus 2 (SARS-CoV-2), primarily affecting the respiratory tract. To date, no vaccines are available nor any specific treatment. To limit the number of infections, strict directives have been issued by governments that have been translated into equally rigorous guidelines notably for post-mortem examinations by international and national scientific societies. The recommendations for biosafety control required during specimen collection and handling have strongly limited the practice of autopsies of the COVID-19 patients to a few adequate laboratories. A full pathological examination has always been considered an important tool to better understand the pathophysiology of diseases, especially when the knowledge of an emerging disorder is limited and the impact on the healthcare system is significant. The first evidence of diffuse alveolar damage in the context of an acute respiratory distress syndrome has now been joined by the latest findings that report a more complex scenario in COVID-19, including a vascular involvement and a wide spectrum of associated pathologies. Ancillary tools such as electron microscopy and molecular biology used on autoptic tissue samples from autopsy are also significantly contributing to confirm and/or identify new aspects useful for a deeper knowledge of the pathogenetic mechanisms. This article will review and summarize the pathological findings described in COVID-19 until now, chiefly focusing on the respiratory tract, highlighting the importance of autopsy towards a better knowledge of this disease.

## Introduction

The viral family Coronaviridae are enveloped, positive-sense, single-stranded RNA viruses, and typically cause mild respiratory diseases in humans [1]. Coronavirus disease 2019 (COVID-19) is caused by the newly emerging severe acute respiratory syndrome (SARS)-related coronavirus species 2 or SARS-CoV-2, which suddenly poses a major health burden to humans. Together with SARS-CoV, the agent of SARS, and MERS-CoV, which causes the middle-east respiratory syndrome (MERS), it represents the third most severe coronavirus disease within the past two decades, currently affecting nearly 8 million patients and with more than 500,000 deaths all over the world. COVID-19 was first recognized in Wuhan, China, in December 2019, wherein a cluster of severe pneumonias occurred in people visiting a seafood and wildlife market [2, 3]. It is thought that this event specifies the origin of the current pandemic, wherein humans potentially acquired the pathogen via animal contact, although newer epidemiological data suggest that the virus might already have circulated earlier. Certain bat species are the natural reservoir for Coronaviridae, but genetic adaptation of the viruses in intermediate hosts seems to play a key role for crossing species barriers and the subsequent transmission to humans. The intermediate hosts for SARS were probably civet cats and raccoon dogs, whereas camels play this role in MERS. In COVID-19, pangolins might have served as such vectors and it is likely that from them, human infection occurred [4]. SARS-CoV-2 infects human cells via binding to the angiotensin-converting enzyme 2 (ACE2) that is highly expressed on epithelial cells of the respiratory tract (e.g., bronchial transient secretory cells), endothelial cells but also on several other cell types [5]. The viral spike (S) glycoprotein, which extrudes from the virion surface, enables attachment to ACE2, and due to cleavage of the S protein by host cell proteases (e.g., via TMPRSS2), fusion of viral and cellular membranes is facilitated to enable viral host cell entry [6]. Thus, the interference of SARS-Cov-2 with multiple physiological processes in several types of cells explains the observed variety of clinical manifestations. Moreover, secondary effects mediated via inflammation and the immune response (e.g., the so-called “cytokine storm”) also seem to contribute significantly to COVID-19 [7]. Clinical manifestations as well show different grades of severity. Risk factors for the development of more severe forms are generally older age and the presence of comorbidities like coronary artery disease, chronic kidney disease, hypertension, obesity, and diabetes type II [8–10]. To that end, there is an urgent need for a better definition of the pathogenetic mechanisms and of the primary and secondary pathologies prevalent in SARS-CoV-2 infection to improve patient care, which could also account for the great variance in the reported case-fatality rates. Such insights might directly enable development of specific therapies to counteract COVID-19.

Awareness about the complexity of this disease has grown over time since its outbreak. Pathological examinations, mainly obtained from autopsy material, have strongly contributed to increase our knowledge of this infection. As expected, the respiratory tract represents the most important target of the disease. Nevertheless, studies focusing on pulmonary pathology are scarce, probably because of the low number of invasive procedures that were performed especially during the first period of the spread. Indeed, the sudden outbreak, the high number of hospitalizations, the shortage of health care personnel, and the high rate of potential contagiousness have limited such procedures. Furthermore, the recommendations for biosafety and infection control required during specimen collection and handling have further affected autopsy practices. To the best of our knowledge, only three papers have focused on histological evaluation of in vivo surgical specimens [11–13] with the main reported pathological findings being diffuse alveolar damage (DAD), organizing pneumonia (OP), reactive type II pneumocytes, and chronic interstitial pneumonia. A more complex scenario is that reported by autopsy studies. This article attempts to provide a comprehensive review of all histological lung lesions reported in the autoptic studies thus far.

## Pulmonary pathology

### The importance of autopsy and biosafety guidelines

The performance of autopsies is widely recognized since many decades as a crucial part of routine pathology practice. However, in the twentieth century, the rate of autopsies decreased significantly, due to factors like improved diagnostic technologies increasing accurate ante-mortem diagnosis, the more complex legislation regarding human tissue examinations, and an insufficient priority given to autopsies by pathologists themselves, struggling with increasing workloads of surgical resections, biopsies, and cytology [14]. Nevertheless, autopsy, followed by microscopic examination of the tissue samplings, still plays a critical role in the diagnosis and in the uncovering the pathophysiology of a newly emerging, yet unknown diseases. Although the declining autopsy rate has been a source of concern to pathologists, clinicians, infectious disease specialists, microbiologists, and epidemiologists increasingly recognize that autopsy is a valuable tool for the evaluation and, ultimately, the control and prevention of emerging and re-emerging infectious diseases [15]. The opportunity of performing post-mortem examinations of patients deceased due to COVID-19 has raised significant concerns motivated by the potential risk of contagiousness. Indeed, the first indications were restrictive and initially discouraged autopsies [16–20]. Surely, the balance of safety and providing quality results is a delicate process that requires strong administration support and leadership. Indeed, both the Centers for Disease Control and Prevention (CDC) and the World Health Organization released documents to provide interim guidelines for the collection, handling, and analysis of clinical specimens that might contain SARS-CoV-2 [21, 22], initially based on previous recommendations for SARS-CoV or MERS-CoV [23]. A crucial point was the risk assessment that has to be conducted in each laboratory, as well as other core processes and procedures that need to be in place to support laboratory biosafety practices when handling a specimen from a patient under investigation for COVID-19 [23–25]. The CDC updated the interim guidelines including specific considerations about the importance, collection, and submission of post-mortem specimens from deceased persons with known or suspected COVID-19 and included specific recommendations for biosafety and infection control practices during autopsy procedures [26].

The College of American Pathologists supported the performance of autopsies in the setting of emerging infectious diseases such as COVID-19 and endorsed the recommendations in the CDC guidelines [27], allowing an increase in number of autopsies performed worldwide and a better knowledge on SARS-CoV-2-related pathology.

There are no guidelines regarding the sampling of whole lungs from autopsy in the literature. Herein, we have reported the lung sampling protocol performed at the University of Padova (see “Autopsy/sampling protocol” section).

### Pathology of lung lesions in COVID deceased patients

**Fig. 1:**
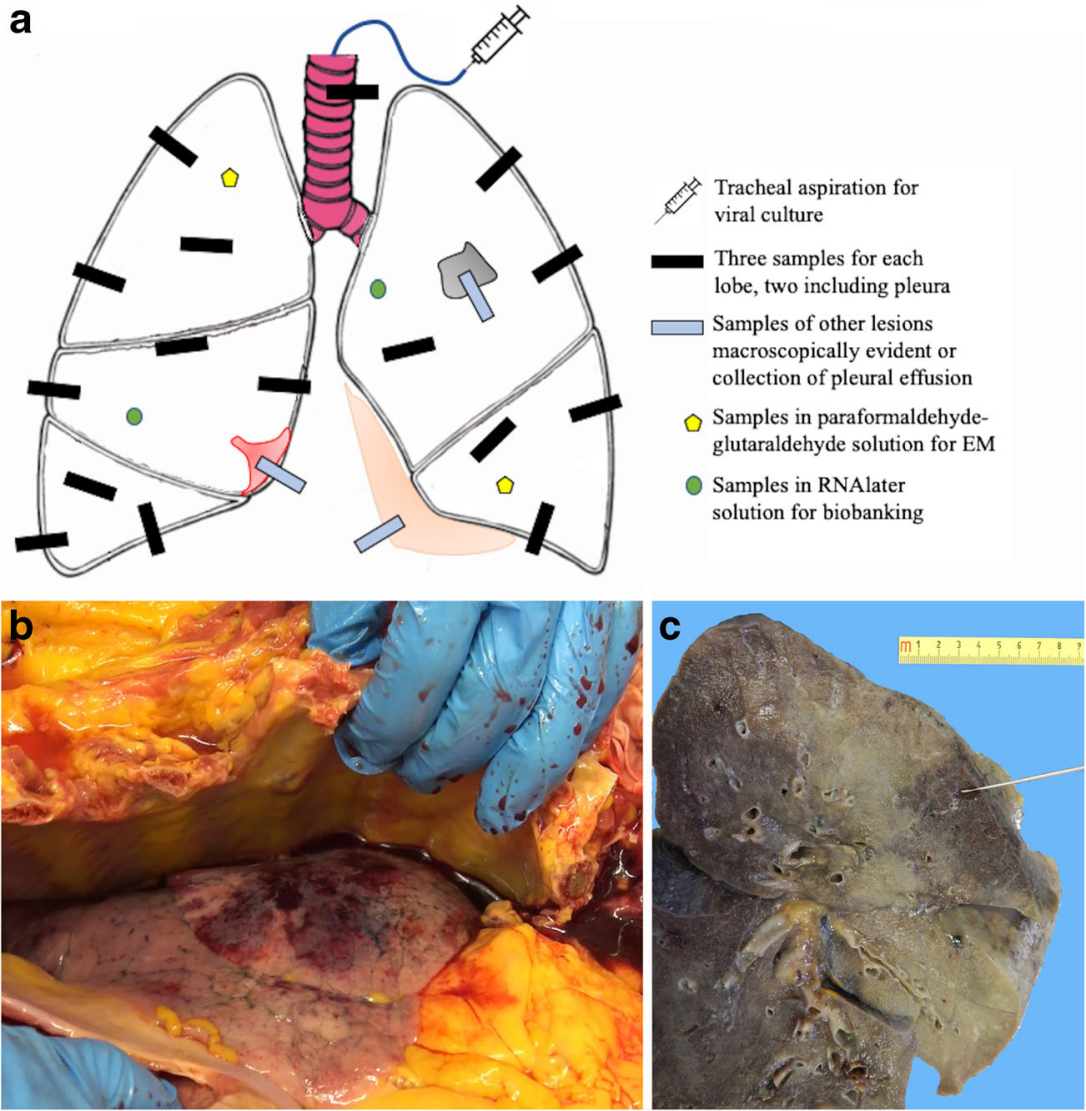
**a** Padova protocol for lung gross examination after sagittal section of lungs: three specimens/lobe (two peripheral and one central) are sampled. When there are other evident lesions or pleural effusions, additional sampling is performed. Transversal section of the trachea and small fragments for cultural, molecular, and ultrastructural analyses are collected. **b** Video frame of the right lung captured during the autopsy in a COVID-19 patient (69-year-old women). A marbled appearance of the lung with bloody pleural effusion was evident. **c** Cut surface of the same lung after formalin fixation. The parenchyma showed patchy areas of consolidation and congestion

The number of published papers in academic journals concerning this field of interest is still scarce. A search in PubMed was done using the keywords “Autopsy and COVID-19”, “Autopsy and SARS-CoV-2”, “Post-mortem and COVID-19”, and the total number at our last review (May 27, 2020) is 23 articles (Table 1, Fig. 1). In the table, we have categorized pulmonary microscopic findings into “alveolar damage”, “vascular injury”, and “airway damage”.

**Table 1:** Articles reporting autopsies in suspected/known COVID-19 patients

First author [Journal]	No cases	Time^a^	Comorbidities (yes/no, details)	Disease duration (days)^b^	Macroscopy	Microscopy		
						Alveolar damage	Vascular injury	Airway damage
Xu Z [Lancet Respir Med] [28]	1	February	No	14	Not reported	Yes	No	Not reported
Tian S [Mod Pathol] [29]	4	March	Yes (CLL, cirrhosis, HTN, DM, renal transplantation)	28	Not reported	Yes	No	Yes
Shao C [Hum Pathol] [30]	1	March	No	25	Not reported	Yes	Yes (microthrombi)	Not reported
Yao X [Cell Research] [31]	1	March	No	16	Not reported	Yes	Yes (microthrombi)	Not reported
Barnes B [J Exp Med] [32]	3	April	Not reported	Not reported	Not reported	Not reported	Yes (capillaritis)	Not reported
Magro C [Transl Res] [33]	2	April	Yes (CAD, DM, HF, obesity)	Not reported	Congestion, hemorrhage	No	Yes (endotheliitis)	Not reported
Barton LM [Am J Clin Pathol] [34]	2	April	Yes (obesity, myotonic dystrophy, HTP)	6	Diffuse edema, pleural adhesions	Yes	Yes (thrombi)	Yes
Varga Z [Lancet] [35]	3	April	Yes (transplantation, CAD, HTP, obesity)	12	Not reported	No	Yes (endotheliitis)	Not reported
Konopka KE [Chest] [36]	1	April	Yes (asthma)	10	Consolidation, mucus plugs	Yes	Yes (thrombi)	No
Copin MC [Intensive Care Med] [37]	6	April	Not reported	12.5	Not reported	No	Yes (endothelial injury)	Not reported
Lacy JM [Am J Forensic Med Pathol] [38]	1	April	Yes (DM, obesity, asthma)	7	Mucus plugs, edema, consolidation, hemorrhage	Yes	No	Not reported
Menter T [Histopathology] [39]	21	May	Yes (HTP, obesity, CVD, DM, immunosuppressed)	6	Consolidation, congestion, suppurative bronchopneumonia	Yes	Yes (vasculitis, microthrombi)	Yes
Wichmann D [Ann Intern Med] [40]	12	May	Yes (obesity, CAD, asthma, COPD, CVD, DM, ND)	Not reported	Congestion, bronchopneumonia, embolism, deep venous thrombosis	Yes	Yes (thrombosis, pulmonary thromboembolism)	Yes
Grimes Z [Cardiovasc Pathol] [41]	2	April	Yes (HTP, HIV)	11.5	Pulmonary thromboembolism, consolidation	No	Yes (pulmonary thromboembolism)	Not reported
Lax SF [Ann Intern Med] [42]	11	May	Yes (HTP, DM, CAD, HL, bladder cancer, COPD, CVD, ND)	8.5	Massive bilateral congestion, mucus plugs, thrombi in branches of the pulmonary arteries, pulmonary infarctions	Yes	Yes (thrombosis of small and mid-sized pulmonary arteries)	Yes
Adachi T [Emerg Infect Dis] [43]	1	May	No	16	Consolidation	Yes	No	Not reported
Yan L [Arch Pathol Lab Med] [44]	1	May	Yes (obesity)	13	Mucous plugs, edema, consolidation	Yes	Yes (vasculitis)	Not reported
Buja LM [Cardiovasc Pathol] [45]	3	May	Yes (obesity, HTP, HF, DM, anemia)	14	Consolidation, congestion, pulmonary thromboembolism, hemorrhage, hyperemic tracheobronchial mucosa	Yes	Yes (pulmonary thromboembolism)	Not reported
Ackermann M [NEJM] [46]	7	May	Yes (HTP, DM, immunosuppression)	Not reported	Weight gain	Yes	Yes (widespread thrombosis, neoangiogenesis)	Not reported
Shaller T [JAMA] [47]	10	May	Yes (HTP, CVD, COPD, DM, obesity, CKD, ND, CMML, CLL, LC)	16	Consolidation	Yes	No	Not reported
Sekulic M [Am J Clin Pathol] [48]	2	May	Yes (HTP, HF, CKD, gout, LC, obesity)	15.5	Bilateral serosanguineous pleural effusion, consolidation, congestion	Yes	No	Yes
Aguiar D [Int J Legal Med] [49]	1	May	Yes (obesity)	Not reported	Consolidation, hemorrhagic edema, pleural effusion	Yes	No	Yes
Fox SE [Lancet Respir Med] [50]	10	May	Yes (HTP, DM, obesity, immunosuppression)	11.6	Weight gain, pulmonary thromboembolism, edema, hemorrhage	Yes	Yes (pulmonary thromboemboli, microthrombi)	No

*ALI* acute lung injury, *CAD* coronary artery disease, *CKD* chronic kidney disease, *CLL* chronic lymphocytic leukemia, *CMML* chronic myelomonocytic leukemia, *COPD* chronic obstructive pulmonary disease, *CVD* cardiovascular diseases, *DM* diabetes mellitus, *HF* heart failure, *HIV* human immunodeficiency virus, *HL* Hodgkin’s lymphoma, *HTN* hypertension, *LC* lung cancer, *ND* neurodegenerative disease

^a^Considered time of manuscript submission

^b^In case series, the mean value of available information was reported

The first autoptic study published by a Chinese group in February 2020 reported a post-mortem minimally invasive core-needle-based tissue collection performed in a 50-year-old man who died from COVID-19. The authors described histological features greatly resembling those seen in SARS and MERS coronavirus infections. Indeed, lung tissue samples showed DAD with cellular fibromyxoid exudates, desquamation of pneumocytes and hyaline membranes, and the main pathological findings of early-phase acute respiratory distress syndrome (ARDS). Moreover, edema, interstitial mononuclear inflammatory infiltrates (mainly lymphocytic), and multinucleated syncytial cells with viral cytopathic-like changes were also present [28]. Other papers published in the same period reported similar histological features [29–31], and in two cases, vascular damage was also described as the presence of microthrombi within pulmonary capillaries associated to the features of acute lung injury [30, 31].

Interestingly, in April 2020, an increasing number of autopsies were performed, representing a crucial turning point in the pathological view of the disease. Indeed, several studies reported not only morphological aspects indicative of ARDS, but overall other lesions, particularly a more consistent description of microvascular injury. Barnes et al. described severe neutrophilic capillaritis in three COVID-19 autoptic patients. Small vessel injury with features of acute capillaritis was found in association with neutrophilic infiltration into the alveolar space and tracheal mucosa. Interestingly, the authors reported a link between the aberrant neutrophilic extracellular traps, so-called “NETs”, and the presence of organ damage both in alveolar parenchyma and airways [32]. Magro et al. reported septal capillary injury accompanied by extensive complement deposition of C4d and C5b-9 in two patients. The authors described thrombogenic vasculopathy also in the skin and notably a colocalization of COVID-19 spike glycoproteins with complement fractions, thus hypothesizing virus-related complement pathway activation [33]. Acute lung lesions and microthrombi were also described in the first report of complete autopsies in two patients who died in Oklahoma (USA) [34]. The authors reported for the first time the feasibility of molecular analysis on post-mortem swabs and other superimposed or unrelated processes. The first clear-cut evidence of viral-related endotheliitis in COVID-19 autoptic samples was reported by Varga et al. The authors demonstrated endotheliitis in different organs (notable, the heart, kidney, lung, small bowel) and aggregates of viral particles with dense circular surfaces and crucial markers in injured endothelial cells. These findings suggested that SARS-CoV-2 infection facilitated the induction of endotheliitis, apoptosis, and pyroptosis in several organs as a direct consequence of the host inflammatory response or, as suggested by some authors, by the direct infection of the endothelial cells [35]. The identification of viral particles in endothelial cells represents a challenging task and a clear-cut evidence is still lacking (see also “Electron microscopy” section). Fibrin thrombi within small vessels and small pulmonary arteries with endothelial damage along with acute lung injury were also reported in the first asthmatic patient who died of COVID-19 [36]. Vascular injury was also consistently detected in six patients who died at different stages of the disease, showing lymphocytic pneumonia and acute fibrinous and organizing pneumonia (AFOP). Copin et al. questioned whether DAD was a frequent injury and observed that there was a more consistent presence of AFOP in severe forms [37]. A sudden death of a 58-year-old female diabetic patient who had extensive lung lesions with diffuse proteinaceous edema, hyaline membranes, prominent desquamating pneumocyte hyperplasia with focal multinucleated cells and bizarre forms was reported [38].

The two largest studies published by Swiss and German groups included 21 and 12 COVID-19 patients, respectively [39, 40]. In the first case series, the primary cause of death was respiratory failure with exudative DAD, massive capillary congestion often accompanied by microthrombi despite anticoagulation and, interestingly, in half of the cases also a superimposed bronchopneumonia [39]. This study provided the first largest overview of post-mortem findings in 21 COVID-19 cases, reporting that hypertensive, elderly, obese, male individuals with severe cardiovascular comorbidities as well as those with blood group A may have a lower threshold of tolerance for COVID-19 [39]. Almost in the same period, Wichmann et al. reported deep venous thrombosis in a high percentage of patients (58%). Venous thromboembolism was not suspected before death and pulmonary embolism was the direct cause of death in four of them, thus suggesting an important role of COVID-19-induced coagulopathy in patient mortality [40]. Pulmonary emboli occluding the main pulmonary arteries were found to be fatal also in two patients described by Grimes et al. who argued that the SARS-CoV-2 infection, like the influenza virus and SARS-CoV, could be associated with coagulation disorders that may lead to thrombosis and disseminated intravascular coagulation [41].

Lax et al. evaluated gross and microscopic findings and clinical correlations in 11 cases. Interestingly, even if ten patients received prophylactic anticoagulant therapy, thrombosis of small and mid-sized pulmonary arteries was found to occur to various extents along with DAD in all of them. The authors suggested that a combination of both lesions (DAD and thrombosis) could explain the rapid clinical deterioration in severe COVID-19, and that more proactive expansion of current anticoagulant strategies is desirable [42].

Adachi et al. reported the autopsy of an 84-year-old cruise ship passenger who died from COVID-19 and lung pathology showed exudative and organizing phases of DAD, similar to that observed in the cases of severe acute respiratory syndrome. By evaluating copy numbers of SARS-CoV-2 by real-time PCR in different specimens, the authors found that the virus principally attacked the respiratory tract and was significantly less present in other organs (brain, heart, testicles, and kidney) and in specimens such as blood, urine, feces, and rectal swab [43].

Yan et al. described an obese female patient of 44 years, showing for the first time an extensive and widespread perivascular lymphocytic cuffing with few foci of infiltration within vessel walls but without fibrinoid necrosis, consistent with non-necrotizing lymphocytic vasculitis. Notably, there was no evidence of microthrombi but ultrastructural investigation revealed fibrin aggregates within blood vessels, suggesting an increased propensity toward clot formation [44]. Autoptic studies of three cases from Houston (USA) showed DAD, diffuse microvascular involvement with intravascular and extravascular fibrin deposition, and diffuse intravascular trapping of neutrophils, hyaline membranes, thromboemboli, and inflammatory, mainly lymphocytic, infiltration. The authors found similar vascular lesions in other organs, supporting the concept that pathogenesis of COVID-19 is related to direct viral injury in different organs with a consequent procoagulant state with coagulopathy [45].

An important study has recently been published by Ackermann et al. [46]. Histological examination of lungs coming from seven deceased patients affected by COVID-19 was compared with that obtained from patients who died from influenza A (H1N1) infection and uninfected control lungs. While DAD with perivascular T cell infiltration was a common feature in SARS-CoV-2 and H1N1 infections, vascular features were instead distinctive of COVID-19 and consisted of severe endothelial injury, widespread thrombosis with microangiopathy, alveolar capillary microthrombi, and neoangiogenesis [46]. Studies that are more recent confirm the complexity of COVID-19, emphasizing the patchy distribution of the disease, mainly focusing on acute lung injury [47–49]. Similar lesions consisting in DAD and diffuse signs of thrombosis and microangiopathy in the small vessels and capillaries of the lungs have recently reported also in a small series of African American community with COVID-19 [50].

Unfortunately, some information is not consistently reported in these studies as, for example, small and large airways as well as pleural tissue. Only nine studies have pointed out some macroscopic and/or microscopic aspects, with pathological ones being mainly related to bronchitis/bronchopneumonia until now [see below for details].

**Fig. 2:**
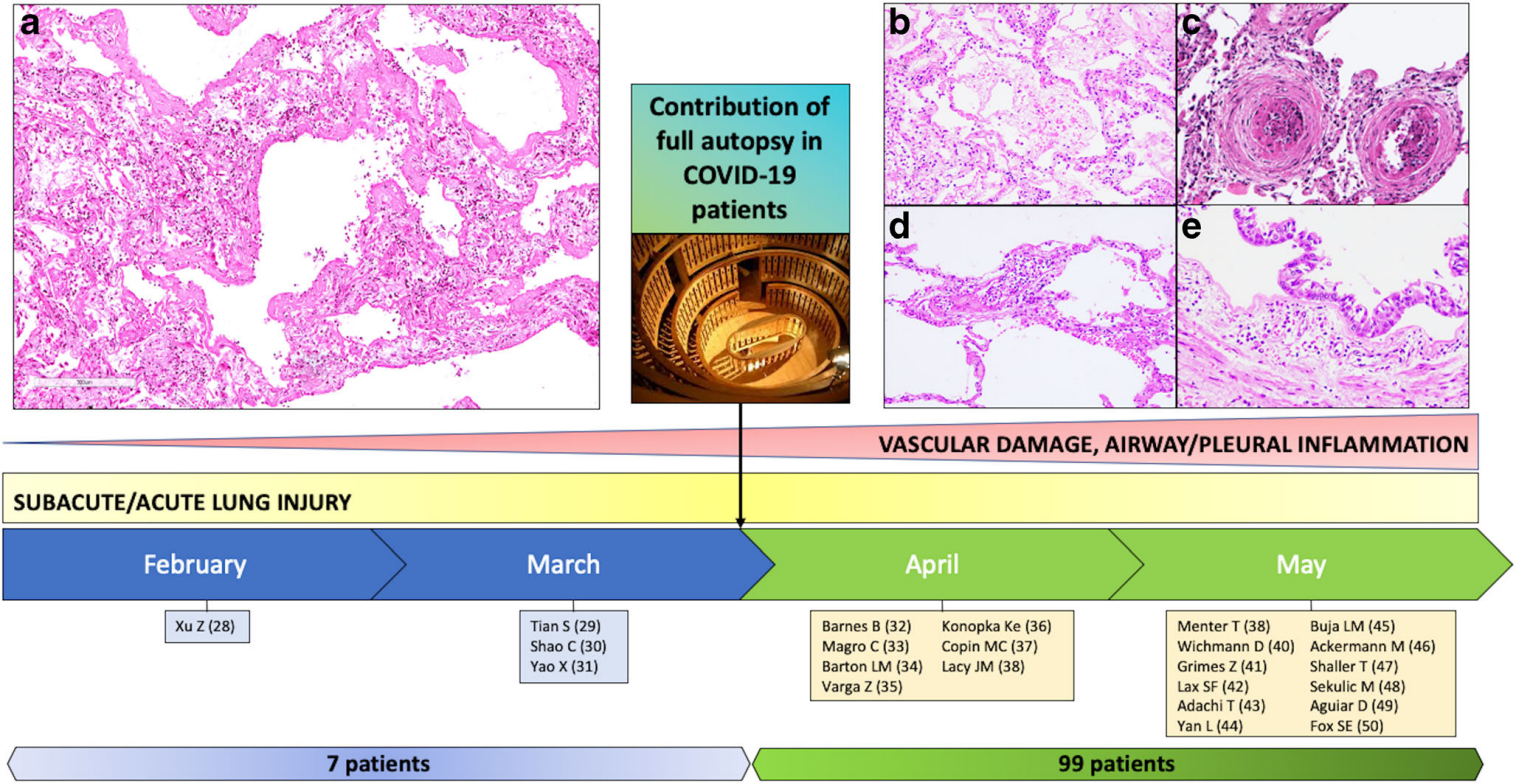
Timeline of autopsy studies focusing on lung lesions in COVID-19 patients. Since the end of March, the increased number of full autopsies has led to a better knowledge of the pathophysiology of the disease. Together with the features of acute lung injury, vascular involvement has been reported. **a**, **b** Acute lung injury: hyaline membrane in alveolar space (hematoxylin and eosin stain, original magnification **a** × 100; **b** × 200). **c**, **d** Vascular damage: two microthrombi in lung small vessels (hematoxylin and eosin stain, original magnification × 200), capillary inflammation (hematoxylin and eosin stain, original magnification × 200). **e** Airway inflammation: tracheal section showing a polymorphous inflammatory infiltrate of the submucosal layers (hematoxylin and eosin stain, original magnification × 200)

Based on a careful revision of autoptic reports, we can distinguish a timeline for the gathering of our knowledge about histological findings: February–March and April–May (Fig. 2). In February–March, four anecdotic case series were published (seven patients in total), and the most described pulmonary pathological lesions were those related to an acute/subacute lung injury. In April–May, 19 articles focused on a more consistent number of autoptic cases (99 patients in total) and it was emphasized that COVID-19 lung lesions included a complex disease involving several anatomic compartments in which the vascular bed was mainly affected.

### Pulmonary and airway-associated lesions

Pulmonary-associated lesions are those unexpected findings that pathologists reported after a careful macroscopic and histological examination of autoptic lung tissue samples. Because autopsies in known or suspected COVID-19 patients have not been performed extensively, to date, limited information is available about pulmonary lesions superimposed to COVID-19 pneumonia. Lesions related to known chronic diseases and comorbidities (such as emphysema, asthma, etc.) will not be considered.

The most frequent finding was superimposed bronchopneumonia (likely bacterial related), both focal and diffuse. In the autoptic case described by Tian et al., there was evidence of consolidation of abundant intra-alveolar neutrophilic infiltration, consistent with bronchopneumonia of a superimposed bacterial infection [29]. Even in the three most numerous case series, bronchopneumonia was found in 33–55% of cases [39, 40, 42]. Minimal submucosal inflammation was described in the bronchi/bronchioles [48, 49], and in two cases, tracheitis was also reported [39, 48]. Even if tracheitis may be explained as an iatrogenic lesion in some patients particularly in those who received invasive ventilation, the finding that these lesions may also frequently occur in patients without invasive ventilation **(**Padova experience, submitted for publication**)** suggests that the trachea is an important target of the disease. This, however, is not an unexpected finding considering that ACE-2 receptors are also expressed in the upper respiratory tract. A recent study supports this hypothesis, documenting important inflammation with an intense scintigraphy uptake on the proximal bronchi in a COVID-19 non-smoker patient [51].

Another important pathological finding was aspiration pneumonia with foreign material, squamous cells, and vegetable matter within the airways [34]. In only one case, lung cancer was reported as an unknown superimposed lesion and it was histologically defined as large cell carcinoma [48]. In our experience, other unexpected pulmonary lesions were also found in COVID-19 autoptic cases, such as lung carcinomas (small cell lung cancer and squamous cell carcinoma), neuroendocrine hyperplasia, aspergilloma, necrotizing granulomas, and pleuro-parenchymal fibroelastosis (unpublished personal data from Padova pathologist team). It may be surmised that more extensive case series could lead to a better understanding of the contributive role of other lesions in the progression and outcome of the disease.

### Comparison of the COVID-19 pandemic and with prior coronavirus pandemics

Few papers have comparatively analyzed COVID-19 pandemic with similar global coronavirus pandemics that occurred over the last decades, such as SARS and MERS. Both diseases had a high mortality and lethality rate. As for the more severe forms of SARS-CoV-2 pneumonias, DAD was reported to be the main histological finding described in the first autopsy case for MERS in the world: the virus showed a tropism for epithelium and was found in pneumocytes and syncytial cells while no evidence of extra-pulmonary involvement was detected [52]. SARS autopsy findings also revealed varying degrees of acute lung injury. The histology varied with the stage of the illness. DAD, bronchiolar fibrin, and airspace edema were mainly detected when the duration of the disease was shorter than 10 days. Organizing pneumonia, type II hyperplasia, squamous metaplasia, multinucleated giant cells, and acute bronchopneumonia were found in longer-lasting diseases [53]. Interestingly, a series of 20 SARS autopsies also showed the involvement of the vascular bed. Indeed, vascular fibrin thrombi, pulmonary infarcts, and small and mid-sized pulmonary endothelial damage were frequently described [54].

In summary, the most severe forms of SARS-CoV-2 certainly share some important similarities with prior coronavirus pandemics. However, COVID-19 has more complex symptoms and progression. The large spectrum of clinical manifestations and degrees of severity have only now been partially explained. Further studies may reveal new insights into the mechanisms of COVID-19.

## Ancillary tools and techniques

Cytology and several ancillary techniques are now considered the routine diagnostic armamentarium in surgical pathology and are particularly helpful in autopsies, especially in case of novel emerging infectious diseases.

### Ancillary tools: cytology

During the COVID-19 pandemic cytology, laboratories are being impacted in several ways [55]. Different respiratory specimens either of SARS-CoV-2 infected or non-infected patients are submitted for cytopathological examination. A cytopathologist may observe morphological features caused by viral infection. Processing of respiratory specimens potentially infected with SARS-CoV-2 requires implementation of biosafety measures in cytology laboratories. The first report on cytopathological features in bronchoalveolar lavage specimens of COVID-19 patients revealed a high number of activated plasma cells admixed with T lymphocytes and scattered B cells [56]. This is a surprising observation since no hyaline material or desquamated pneumocytes were found which would have been expected based on the main histopathological changes reported in COVID-19 lungs. SARS-CoV-2 infection does not cause any specific cytomorphological features. In various respiratory specimens, like sputum or bronchoalveolar lavage, we might observe cytomorphological features of acute lung injury and repair: increased number of macrophages, atypical type 2 pneumocytes, squamous metaplastic cells, and multinucleated cells. Viral etiology might be suspected based on cytoplasmic and nuclear changes in macrophages and epithelial cells. Presence of cell and nuclear enlargement, epithelial desquamation, foamy cytoplasm, larger paranuclear cytoplasmic vacuoles, nuclear clearing, intranuclear inclusions, all of which might be related to viral infection, may represent a potential diagnostic pitfall [55]. Indeed, routine laboratory processing pleural effusion, bronchial washings and aspirates, bronchoalveolar lavage, and rinsed transbronchial needle aspiration specimens need centrifugation and/or cytocentrifugation which produces aerosol and causes the most potentially infectious working exposure. Strict biosafety measures should be implemented to prevent accidental or unintentional exposure to infection [55, 57]. Several guidelines have been published to help health care workers operate safely in cytology laboratories [21, 58]. Concerning autoptic reports, a post-mortem cytological investigation of SARS-CoV-2 infection was performed only in very few cases. Barton et al. first reported viral positivity in post-mortem nasopharyngeal and bilateral lung parenchymal swabs [34]. Moreover, a forensic case of a sudden unexpected death was related to COVID-19 after analyzing dacron-tipped swabs of the right and left main bronchi [38]. More recently, also other cases have been investigated in post-mortem cytological samples, not only in swabs [43, 45, 47, 49] but also in pleural effusions [47].

**Fig. 3:**
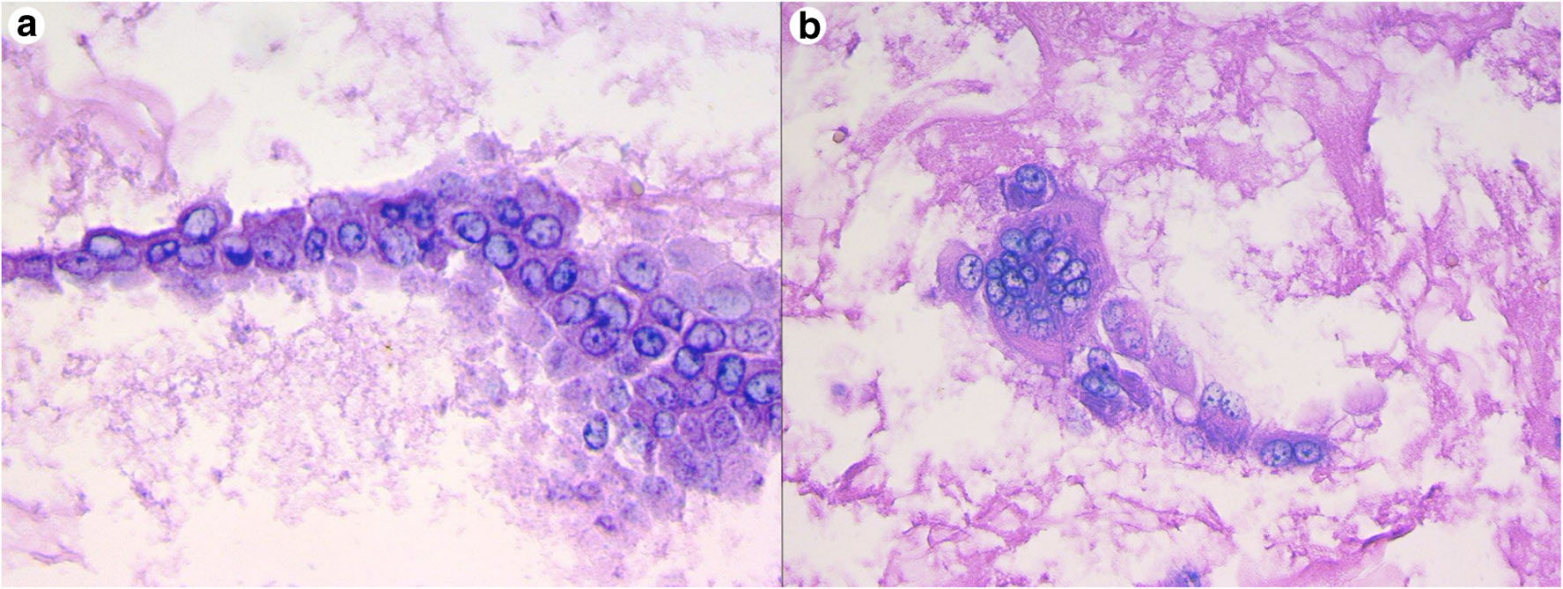
Cyto-block preparation of pleural effusion fluid. Aggregates of dysmorphic mesothelial cells with enlarged nuclei (**a** hematoxylin and eosin, original magnification × 600) and multinucleated syncytial cell (**b** hematoxylin and eosin, original magnification × 400). These features suggest viral infection, as was confirmed by the positivity of RT-PCR for SARS-CoV-2 on the cytological specimen

Pleural effusions were radiologically and/or grossly mentioned in different studies on autoptic cases [42, 43, 45, 47, 50]. In particular, when case series were reported, the prevalence of pleural effusions was either relatively small (9% and 33%) [42, 45] or was not specified [47, 50]. In our experience (University of Padova), pleural effusion was detected in more than half the patients (59% of cases). We collected it when it was more than 200 cc. When a formalin-fixed cell pellet was processed for cytoblock evaluation, several reactive mesothelial cells in a mild inflammatory background were present (Fig. 3).

The value of cytological examination in COVID-19 may be limited. Given its scarce contribution to the diagnosis and the difficulties in sample management, several limitations remain unsolved especially in relation to the real risk-benefit ratio.

### Ancillary techniques

#### Electron microscopy

**Fig. 4:**
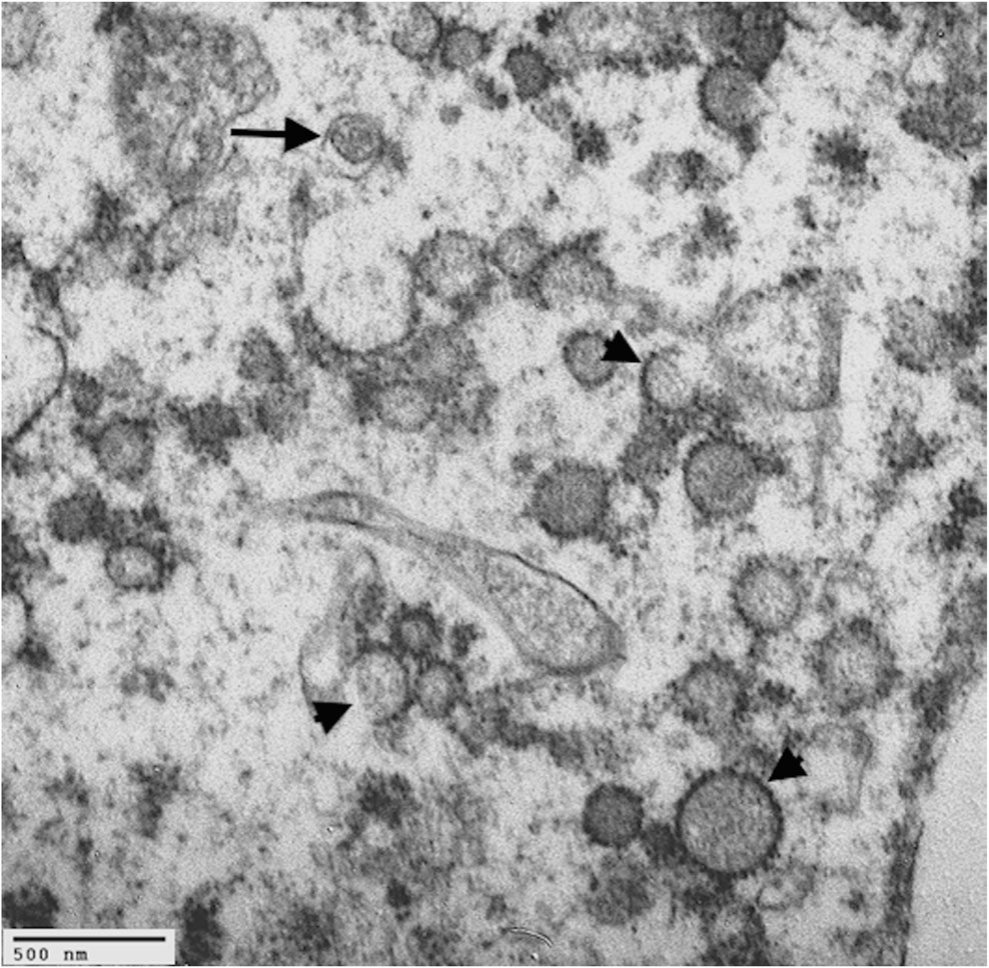
Cytoplasm of type II pneumocyte from a post-mortem lung sample of a COVID-19 patient. The molecular test RT-PCR for SARS-CoV-2 was positive on lung tissue. Post-mortem autolytic phenomena prevent a precise visualization of sub-cellular compartments. There are several spherical particles outlined with electron-dense dots that could mimic coronavirus-like virions. Most probably, some of these particles are clathrin-coated intracytoplasmic vesicles (arrowheads) or cross-sections of the rough endoplasmic reticulum. Immunogold labeling would be desirable to verify the nature of these putative viral particles. A microvesicular body/autophagosome was also evident (arrow) (transmission electron microscopy, original magnification × 30,000)

Electron microscopy (EM) represents an important ancillary technique and provides important insights in COVID-19 lung tissue including autopsy material. Transmission EM (TEM) could be particularly useful for identifying SARS-CoV-2 in specific cells or subcellular compartments. Almeyda and Tyrrell first described the characteristic ultrastructural morphology of coronavirus particles in 1965 from organ cultures of respiratory epithelium [59]. The virions were roundish, ranging from 80 to 120 nm in diameter, and with 20-nm long tail-like projection. Subsequent electron microscopy studies carried out on fetal lung cell cultures have more fully investigated the morphological characteristics of the virus [60]. The spherical particles filled the cisternae of the endoplasmic reticulum of infected cells mostly in perinuclear areas and were present in the extracellular spaces following the cell lysis. SARS-CoV-2 shares the morphological features of the Coronaviridae family [61]. The first ultrastructural in vivo study of SARS-CoV-2 in lung was performed on bronchoalveolar lavage fluid samples [62]. The authors demonstrated the presence of cytoplasmic inclusion bodies containing the typical virions only in the respiratory epithelial cells. Since then, several autopsy studies have consistently used TEM to assess the presence of SARS-CoV-2 particles in different tissues and cell types. Experimentally, it has been observed that the virus can infect engineered human blood vessel organoids and human kidney organoids via the ACE-2 pathway [63]. This observation, together with the frequent findings of vascular damage, has focused the research of the virus in vascular tissues. Some authors reported the presence of putative SARS-CoV-2 particles in the cytoplasm of endothelial cells [35, 40] but these observations have been questioned by experts [64]. TEM can be a powerful tool to find evidence of infection by a virus, but care must be taken when interpreting unequivocally such cytoplasmic structures as viral particles. There are numerous structures found by TEM that resemble viruses (the so called viral-like particles), such as endothelial tubuloreticular inclusions, clathrin-coated vesicles at the trans-Golgi-network, multivesicular bodies/autophagosomes or, straightforwardly, cross-sections of the rough endoplasmic reticulum (Fig. 4). The correct interpretation is even more difficult when the ultrastructural study is carried out on autoptic samples usually affected by several artifacts due to autolytic processes or inadequate fixation. More advanced techniques, as the immune-gold EM, are required to discern with certainty whether these structures are SARS-CoV-2 virions. The study of vascular alterations in COVID-19 has also been conducted by scanning electron microscopy (SEM) analyses. Ackermann et al. [46], using the microvascular corrosion casting method, highlighted the architectural distortion and the loss of a clear vessel hierarchy of the alveolar vascular plexus secondary to intussusceptive angiogenesis. The authors showed a significantly greater density of intussusceptive angiogenic features in COVID-19 lungs as compared to influenza A and the control group. The results obtained from the ultrastructural analyses of COVID-19 in the lungs are still limited. A more extensive use of TEM, immunogold labeling, or SEM may be appropriate to have a better understanding of some altered subcellular structures.

#### Other tissue techniques

Outside the TEM approach, different tools have already been used for many years to identify different coronavirus types in human diseases such as those due to the MERS coronavirus and the SARS-CoV, from formalin-fixed paraffin-embedded (FFPE) tissue specimens, notably obtained from autopsies [52, 65]. These tools include immunohistochemistry (IHC) and in situ hybridization (ISH) as well as molecular biology using real-time reverse transcriptase (RT) polymerase chain reaction (PCR)-based assay from tissue sections and were thus developed to identify the SARS-CoV-2 in FFPE tissue specimens too. So far, during the COVID-19 pandemic, a few studies have been published showing the usefulness of IHC and ISH as well as RT-PCR to detect the SARS-CoV-2 from FFPE tissue specimens, but rarely from samples taken during autopsies [11, 31, 66, 67]. For a pathologist point of view, IHC detection of SARS-CoV-2 virus in lung specimens is the best way to combine etiology with morphological changes. Liu et al. have recently used different antibodies to look for the SARS-CoV-2 in infected FFPE cell pellets [66]. These antibodies included a rabbit polyclonal anti-SARS-CoV-2 spike protein antibody and a mouse monoclonal anti-SARS-CoV-2 nucleocapsid protein antibody. The authors also developed RNAscope ISH to identify SARS-CoV-2 RNA. Additionally, they developed a dual staining assay using both IHC and ISH to detect SARS-CoV-2 protein and RNA in the same FFPE cell pellet sections [66]. This latter approach using dual staining is certainly very interesting because a positive IHC and RNA signal alone to detect the SARS-CoV-2 may originate from degenerating RNA fragments or remaining free viral antigens and not from viral particles. These approaches need to be validated now in FFPE tissue sections from patients infected by SARS-CoV-2, notably in samples taken during autopsy. Using IHC with anti-Rp3 NP protein of SARS-CoV-2, a study conducted by Zhang et al. using tissues collected transthoracically with a minimally invasive core needle revealed that the virus was present mainly in alveolar epithelial cells [11]. Another study conducted by Yao et al. detected SARS-CoV-2 nucleocapsid in post-mortem lung tissue in both bronchiolar and type II alveolar epithelial cells using monoclonal antibody against SARS-CoV-2 nucleocapsid. It is noteworthy that this latter IHC was negative in different post-mortem tissues including the heart, intestine, skin, liver, and bone marrow [31]. The same antibody was used in other studies [43] as well as for immunofluorescent microscopic visualization of infected cells in bronchoalveolar lavage specimens [3].

During the COVID-19 pandemic, SARS-CoV-2 has also been identified by molecular biology using RT-PCR-based assays in many human samples including bronchoalveolar lavage, sputum, nasal swabs, bronchial brush biopsy, feces, and blood [68]. It is noteworthy that RT-PCR can be used to detect SARS-CoV-2 RNA in deparaffinized tissue sections from different FFPE tissue samples as well [11]. RT-PCR was also performed in autopsy samples [29, 40, 43]. One problem with molecular analyses is the PCR performance with the risk of false negative and/or false positive results in suspected cases with typical COVID-19 clinical/radiological characteristics [69–72]. There may also be problems in the sampling, such as RNA degradation when storage systems are inadequate. The protocol employed should be optimized for FFPE tissue and the use of a kit designed for other purposes should be avoided. Moreover, quantitative PCR assay does not always allow for the discrimination between genomic and subgenomic RNA, which contrasts with the important need to assess viral replication.

The CDC and Prevention has designed a SARS-CoV2 RT PCR diagnostic panel to minimize the chance of false positive results (https://www.cdc.gov/coronavirus/2019-ncov/lab/rt-pcr-detection-instructions.html). This highlights the mandatory necessity to be aware of using adequate positive and negative controls in carrying out molecular tests for detecting SARS-CoV2 from FFPE autopsy samples. In a very recent paper, RT-PCR has been used with good results in FFPE specimens obtained from several major organs, giving important insights in the pathophysiology of the disease [48]. The authors also performed next-generation sequencing from post-mortem tissue samples identifying a mutation that was quite consistent with a subset of the Western European Clade A2a [48], overcoming the limits reported by different papers about the use of such molecular investigations in post-mortem tissue samples.

## COVID-19 multiorgan involvement

Although the respiratory tract is the main target of COVID-19, some clinical evidences suggest extra-pulmonary involvement. Some case-series studies have emphasized that the renal, cardiac, nervous, cutaneous, and gastrointestinal manifestations which occur during the disease may be related to SARS-CoV-2 infection [73]. It remains unclear, however, if these manifestations are directly caused by infection of SARS-CoV-2, or to secondary phenomena like inappropriate or overwhelming immune responses, treatment effects or ischemia due to respiratory impairment or thrombosis. A few authors have quantified viral load in several types of tissues, finding lower levels of SARS-CoV-2 in the kidneys, liver, heart, and brain [74], thus supporting secondary rather than primary involvement. This possibility might be justified by the ubiquitous expression of ACE2 and would be in line with the hypothesis of the multiorgan tropism of SARS-CoV-2.

## Autopsy/sampling protocol

Postmortem examination of COVID-19 should be performed in an autopsy suite, equipped if possible with a ventilation system with six complete air changes/h (ACH) in a pressure-negative environment, with air exhausted through HEPA filters [Biosafety Level 3 (BSL3)]. A detailed autopsy/sampling protocol has recently been published by the University of Padova Pathological Section [75]. The protocol requires that a video registration be taken during the autopsy. The gross examination and sampling of the whole lung is carried out in the same manner as that used for explant lung evaluation. After 72 h, the lungs can be weighed, sectioned, and sampled in a total number of at least 16 samples (three samples per lobe + one at the hilum) for the lungs. Sampling is also carried out when other lesions are present and are clearly visible during the examination (Fig. 1).

## Conclusions

Using invasive diagnostic procedures to obtain tissue specimens from COVID-19 patients is often not feasible considering the critical conditions of these patients with a high mortality risk and the often-sudden clinical presentation. Information coming from the most recent autopsy studies has been crucial and has marked an important step forward to gain better knowledge of the pathological substrates of COVID-19.

## Data Availability

Not applicable.
